# RETINAL AGING IN COMPANION DOGS

**DOI:** 10.1093/geroni/igad104.1481

**Published:** 2023-12-21

**Authors:** Freya Mowat, Michele Salzman, Natascha Merten, Callie Rogers

**Affiliations:** University of Wisconsin-Madison, Madison, Wisconsin, United States; University of Wisconsin-Madison, Madison, Wisconsin, United States; School of Medicine and Public Health, University of Wisconsin, Madison, Wisconsin, United States; University of Wisconsin-Madison, Madison, Wisconsin, United States

## Abstract

Companion dogs cohabit with humans in approximately 45% of US households. They experience similar environmental exposures and lifestyles to their human companions, and have shown sensory, cognitive, and neurological decline with aging. The relationship between age and retinal neurologic function in dogs has not been described. The aim of this study was to determine associations between age and retinal full-field electroretinographic measures in companion dogs, an important translational model species for human neurologic aging. Healthy adult companion dogs with no ophthalmic abnormalities were included (n=54, 18 male, 36 female, median weight 25.7kg, median age 89 months). Unilateral full-field light-adapted and dark-adapted electroretinography (ERG) was performed using a handheld device, with mydriasis and topical anesthesia. Standard least squares effect leverage models were used with log transformed data to determine the cross-sectional effect of age, sex, and bodyweight on ERG amplitudes and peak times. Age and sex were significantly associated with ERG outcome variables, more consistently with ERG amplitude than peak time; age had the largest and most significant effect leverage on outcomes (significantly affecting 3/9 peak time and 8/10 amplitude outcome variables). Younger and female dogs had higher ERG amplitudes. Body weight had no significant effect on any outcome. Our finding that aged companion dogs have reduced ERG amplitudes and prolonged peak times mirror ERG findings in aging humans. Because dogs have a shorter lifespan compared with humans, they may act as a sentinel species to monitor for emerging risk factors for age-related neurologic decline.

